# NanoArrayPAD−X: Nanoprobe Array and 3D-µPAD for the Simultaneous Detection of Respiratory Pathogens and Biomarkers at the Point of Care

**DOI:** 10.3390/bios15110715

**Published:** 2025-10-28

**Authors:** Andreu Vaquer, Francisco M. Bouzada, Sofia Tejada, Antonio Clemente, Antonia Socias, Maria Aranda, Alberto del Castillo, Joana Mena, Maria Montaner, Rocío Rodríguez, Estrella Rojo-Molinero, Antonio Oliver, Marcio Borges, Roberto de la Rica

**Affiliations:** 1 Multidisciplinary Sepsis Group, Health Research Institute of Balearic Islands (IdISBa), Son Espases University Hospital, 07120 Palma de Mallorca, Spain; 2 Department of Chemistry, University of the Balearic Islands, 07122 Palma de Mallorca, Spain; 3 Group of Innovation in Immunopathology of Infections (GTERi), Health Research Institute of the Balearic Islands (IdISBa), 07120 Palma de Mallorca, Spain; 4 CIBER de Enfermedades Infecciosas (CIBERINFEC), Instituto de Salud Carlos III, 28029 Madrid, Spain; 5 Multidisciplinary Sepsis Unit, Son Llàtzer University Hospital, 07198 Palma de Mallorca, Spain; 6 Microbiology Department, Health Research Institute of Balearic Islands (IdISBa), Son Espases University Hospital, 07120 Palma de Mallorca, Spain

**Keywords:** VAP, pneumonia, bacteria, biosensor, multiplexed, paper-based

## Abstract

Microfluidic paper-based analytical devices (µPADs) are ideal for point-of-care diagnostics due to their low cost, compact size, and ease of use. However, current designs have limited multiplexing capabilities, making it difficult to simultaneously detect pathogens and biomarkers in the same sample. In this work, we introduce NanoArrayPAD−X, a novel µPAD design that combines wax-printed microfluidic networks with an array of nanoprobes for the simultaneous detection of multiple targets. This is achieved by distributing the sample through the microfluidic network containing X detection areas. There, targets are captured through physical interactions and recognized by specific antibody-coated nanoprobes released from the nanoprobe array. This generates X dots whose color depends on the concentration of the targets in the sample. A NanoArrayPAD−5 platform capable of detecting five targets was developed to aid in the diagnosis of ventilator-associated pneumonia (VAP). The sensor array could detect *Pseudomonas aeruginosa*, *Klebsiella pneumoniae*, *Staphylococcus aureus*, *Escherichia coli*, and the inflammatory biomarker myeloperoxidase (MPO) with a total turnaround time of 25 min, which is faster than waiting for an overnight culture and the results of an ELISA. Notably, our prototype successfully detected the targets in 87 bronchial aspirate (BAS) specimens, thus demonstrating the suitability of the platform for analyzing complex samples with sputum-like qualities. These findings establish NanoArrayPAD−X as a promising tool for the rapid, multiplexed screening of respiratory pathogens and biomarkers, with potential for guiding personalized antimicrobial therapy in suspected cases of nosocomial pneumonia.

## 1. Introduction

Bacteria in the Intensive Care Unit (ICU) have evolved multiple mechanisms of antimicrobial resistance due to the intensive use of antibiotics in this setting [1]. This issue, combined with a patient population that is often immunocompromised, makes lower respiratory tract infections a major health concern in the ICU [1]. Patients on mechanical ventilation are particularly vulnerable, since the catheter used for intubation can be colonized by bacteria and lead to ventilator-associated pneumonia (VAP), a serious complication that significantly increases morbidity and mortality [2]. Identifying VAP rapidly and providing adequate antibiotics is key to improving outcomes. This is currently challenging because available diagnostic approaches take too long to guide initial patient management. For example, the evaluation of symptoms and radiologic findings often fails to diagnose VAP early and with high accuracy [3]. Respiratory biomarkers are not used in standard clinical practice because it is difficult to quantify their levels in lower respiratory tract specimens using conventional ELISA [4]. Detecting the presence of potentially harmful bacteria by culturing respiratory samples requires more than 48 h, which makes it impractical for the early diagnosis of VAP [5]. Bacterial identification can be expedited by using PCR panels such as Biofire FilmArray, which detect pathogens and genes associated with antibiotic resistance within an hour [6,7]. While these tools offer high sensitivity and diagnostic accuracy, their widespread use is limited by high costs and the requirement for bulky equipment. Furthermore, they are not available at the point of care, which further delays the diagnosis. In this context, a point-of-care test capable of detecting respiratory biomarkers and pathogens could help diagnose VAP rapidly, ensuring that patients receive adequate treatments from the onset and improving patient prognosis.

Three-dimensional microfluidic paper-based analytical devices (3D-µPADs) have emerged as a promising technology to address some of these challenges [8,9,10,11]. Paper naturally wicks liquids, enabling the creation of microfluidic systems by defining channels with water-resistant materials such as wax. This technology facilitates liquid handling, thus making the analysis better suited for point-of-care diagnosis. Combining this method with colorimetric transduction mechanisms [12], which can be interpreted by eye or with a smartphone app, results in biosensors that can be easily implemented without using additional instrumentation. In the field of infectious disease diagnosis, a 3D-µPAD capable of identifying different Dengue virus serotypes [13] has been proposed. However, the device has not yet been tested on respiratory specimens, and its effectiveness in detecting both pathogens and biomarkers within the same sample remains to be demonstrated. Other approaches are not suitable for point-of-care testing. For instance, a 3D-µPAD immunosensor for detecting *Plasmodium falciparum* histidine-rich protein 2 used ambient mass spectrometry [14], which requires bulky equipment that is difficult to implement by the bedside.

The solution to this issue may lie in the technology behind reverse-phase protein lysate microarrays [15,16], since this approach facilitates the detection of multiple targets simultaneously. In reverse-phase microarrays, cells are lysed, and the resulting mixture of biomolecules is adsorbed onto a substrate. Then, an array of labeled antibodies is dispensed for recognizing multiple analytes simultaneously. This creates a matrix of dots, which are usually detected with fluorescence-based methods. While this approach could enable the simultaneous detection of protein biomarkers and pathogens using an array of antibodies, it is restricted to well-equipped laboratories with the right infrastructure for producing and analyzing microarrays. Therefore, the requirement of bulky instrumentation limits their applicability at point-of-care settings where both equipment availability and space are limited.

**Figure 1:**
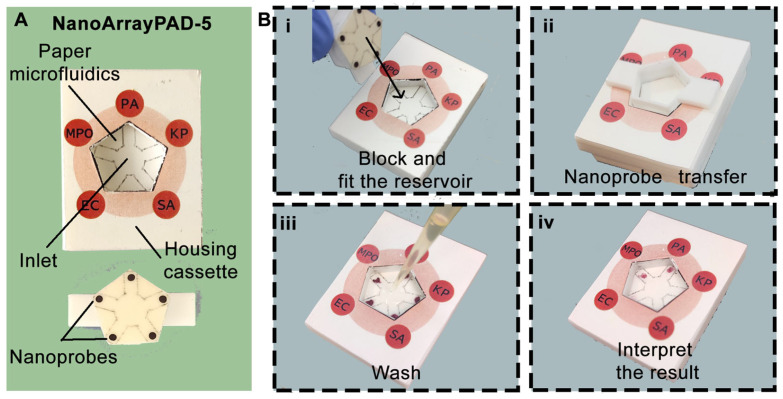
Images of the NanoArrayPAD−5 and sample analysis workflow. (**A**) Device overview and components: sample pad with microfluidic system and fitting piece containing nanoprobe arrays. (**B**) Detection steps: (**i**) sample and blocking solution addition; (**ii**) nanoprobe transfer to detection areas; (**iii**) washing off excess nanoprobes; (**iv**) color intensity quantification to assess infection risk. PA: *Pseudomonas aeruginosa*; KP: *Klebsiella pneumoniae*; SA: *Staphylococcus aureus*; EC: *Escherichia coli*; MPO: myeloperoxidase.

To address the lack of rapid and multiplexed diagnostic tools for ventilator-associated pneumonia (VAP), we developed NanoArrayPAD−X, a novel platform that integrates the high multiplexing capability of reverse-phase protein arrays with the simplicity of paper-based colorimetric detection. As illustrated in Figure 1A, the device enables simultaneous detection of four bacterial pathogens and one respiratory biomarker using a wax-printed microfluidic system and an array of antibody-coated nanoprobes. This combination allows straightforward sample handling (one-minute liquefaction step), minimal instrumentation, and analysis within 25 min, making NanoArrayPAD−X a promising approach for point-of-care diagnosis of VAP. Steps involved in the assay performance are illustrated in Figure 1B(i–iv).

## 2. Materials and Methods

### 2.1. Materials

SYTO9 dye and 1-Ethyl-3-(3-dimethylaminopropyl) carbodiimide (EDC) were purchased from ThermoScientific (Waltham, MA, USA). Whatman filter paper #41 and #1 were obtained from Cytiva (Uppsala, Sweden). Albumin from bovine serum (BSA, protease free) was obtained from VWR Chemicals (Radnor, PA, USA). Commercial plastic housing cassettes were purchased from MDI Advanced Microdevices (Ambala Cantt, India). Rabbit anti-Pseudomonas aeruginosa (PA) polyclonal antibody biotin, Rabbit anti-E. coli (EC) serotype O/K polyclonal antibody biotin and Rabbit anti-Klebsiella pneumoniae (KP) polyclonal antibody biotin were purchased from Invitrogen (Waltham, MA, USA). Rabbit anti-Staphylococcus aureus (SA) polyclonal antibody was purchased from Abcam (Cambridge, UK). Rabbit anti-myeloperoxidase (MPO) polyclonal antibody was purchased from Bioss Antibodies (Woburn, MA, USA). Human myeloperoxidase, Gold (III) chloride trihydrate, sodium citrate tribasic dehydrate, poly (ethylene glycol) 2-mercaptoethyl ether acetic acid (thiol-PEG-acid) 2100, glycine, N-Hydroxysulfosuccinimide sodium salt (sulfo-NHS), Poly (ethylene glycol) 2-aminoethyl ether biotin (PEG-biotin), hydrogen peroxide 30%, 30% poly (sodium 4-styrenesulfonate) solution (average Mw ≈ 70.000), avidin from egg white, Tween-20, and Luria–Bertani medium (LB) were purchased from Merck/Sigma-Aldrich (Darmstadt, Germany). PBS refers to phosphate-buffered saline pH 7.4. PBST refers to phosphate-buffered saline pH 7.4 doped with 0.1% of Tween-20.

### 2.2. Methods

#### 2.2.1. Manufacturing of NanoArrayPAD−X

Housing cassettes and their fitting pieces were designed using Tinkercad (version 1.4) and manufactured using a Prusa Mini 3D printer (Prusa research, Prague, Czech republic) (See Figure S1). Paper templates for sample pads and for nanoprobe arrays were designed using Photoshop (version 20.0.5). Then, the patterns were printed onto Whatman filter paper #41 and #1, respectively, using a Brother MFC-1910W printer (Brother industries, Nagoya, Japan). Paper microfluidic systems were obtained by imprinting barriers using a home-made wax pencil, which was subsequently infused into the paper matrix by placing the paper substrate on a hot plate for 20 s (Figure S2A). The wax-patterned paper piece was then folded and placed into the housing plastic cassettes along with 8 pieces of 2.5 × 2.5 cm^2^ of blotting paper. The array of nanoprobe reservoirs was fabricated using a previously published protocol [17]. Briefly, a volume of 0.5 µL of nanoprobes modified with target specific antibodies were manually spotted using a Hamilton syringe (Hamilton company, Bonaduz, Switzerland) on poly (sodium 4-styrenesulfonate) paper films. The detailed assembly of NanoArrayPAD−X platform is detailed in Figure S2B. The average humidity in the laboratory was 55.5%.

#### 2.2.2. Detection of Bacteria and MPO

The synthesis of gold nanoprobes (AuNPs) and modification with antibodies were performed following a previously published protocol [18]. The procedure is detailed in Section S1. Sample volume optimization before applying NanoArrayPAD−5 to bacterial detection is detailed in Figure S3. Since the specific detection of the target analytes is achieved by immunodetection, the performance of AuNPs functionalized with antibodies against bacteria was evaluated by performing calibration experiments using solutions with increasing concentrations of bacteria in the range from 10^3^ to 10^8^ CFU·mL^−1^ in PBS. UV–Vis spectra of the nanoprobes at different stages of modification are shown in Figure S4A. Negative control with no bacteria was also included. Bacterial suspensions were adjusted to an optical density of 1 a.u. measured at 600 nm in LB medium. It was assumed that this optical density corresponds to 10^8^ CFU·mL^−1^. The specificity was evaluated by testing the nanoprobes against increasing concentrations of bacteria different from the target one. The performance of AuNPs coated with anti-MPO antibody (α-MPO) was evaluated by performing a calibration experiment using solutions with increasing concentrations of MPO in PBS in the range from 0.03 to 30 µg·mL^−1^. A negative control with 0 µg·mL^−1^ of MPO was also included. Nanoprobe optimization and cross-reactivity experiments were performed as single-strip assays, following a previously described protocol. Briefly, 20 µL of sample was added to a 2 × 8 cm^2^ Whatman #41 paper strip. After drying, the strip was blocked with PBS-BSA, and the nanoprobe reservoir was pressed against the sample pad to transfer the AuNPs. After 5 min, excess nanoprobes were washed with PBST, and the resulting colored dots were scanned using a Brother MFC-1910W scanner and the pixel intensity quantified as detailed below. All the experiments described in this section were performed in triplicate.

#### 2.2.3. Quantification of Colorimetric Signals

The images of scanned paper substrates were analyzed using the ImageJ software (version 1.53K). Briefly, the pixel intensity (PI) of the region of interest was measured in the grayscale channel. Then, the S signals were obtained by subtracting the PI from 255. A schematic representation of the quantification process can be found in Figure S4B.

#### 2.2.4. Processing and Analysis of BAS Samples

In total, 87 bronchial aspirate samples (BAS) were obtained from patients admitted to the ICU of Son Llàtzer University Hospital (HUSL, Balearic Islands) from March to December 2023. All patients were mechanically ventilated when the samples were collected.

The BAS samples were obtained by daily aspiration of mucus from the trachea and bronchi. The process is performed as routine clinical practice in patients with mechanical ventilation (Ethics and Scientific Committee approval with reference: IB 4988/22 PI). Samples were sent to the Department of Microbiology at Son Espases University Hospital (HUSE, Balearic Islands), where they were divided into two aliquots. The first aliquot was destined for microbial culture, and the other was stored at −80 °C until it was used for detecting bacteria and biomarkers using NanoArrayPAD−5.

The Department of Microbiology reported a result of semiquantitative microbiological culture as a “positive/negative culture”, and the pathogen isolated was informed. For the detection of bacterial pathogens in real BAS samples, the samples were stratified as follows: target pathogen group, which included those where one of the pathogens of interest (PA, KP, SA, or EC) was isolated with a bacterial count ≥10^5^ CFU·mL^−1^ (clinical threshold for bacterial infection), and control group, comprising samples with non-target pathogens, no microbial growth, or mixed flora (multiple species grew but none predominated over the others). To assess the capability of MPO to screen for early stages of potential lower respiratory tract infections, the samples were classified into two groups: positive (indicating the isolation of a potentially pathogenic microorganism) and negative (no growth observed in the culture medium or mixed flora).

A previously described protocol [19,20] based on activating catalase was used for liquefying BAS samples. Briefly, BAS samples were weighed out in a conical 15 mL polypropylene tube and liquefied by adding 0.3 M H_2_O_2_ in PBS at a 20:1 constant ratio (*v*/*w*) for 2 min at room temperature.

## 3. Results and Discussion

**Figure 2:**
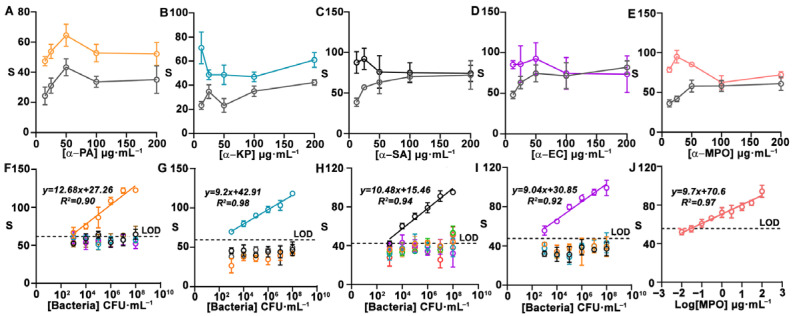
Nanoprobe optimization and cross-reactivity studies. Colorimetric signal for detecting PA (**A**)**,** KP (**B**), SA (**C**), and EC (**D**) at 0 (gray) and 10^7^ CFU mL^−1^ (orange, blue, black, and purple), and MPO (**E**) at 0 (gray) and 3 μg mL^−1^ (pink) using nanoprobes modified with target-specific antibodies at different concentrations. Calibration plots for single targets at different concentrations (orange: PA; blue: KP; black: SA; purple: EC; pink: MPO; brown: *S. maltophilia*; green: *S. pneumoniae*; and red: *S. epidermidis*) obtained with nanoprobes against PA (**F**), KP (**G**), SA (**H**), EC (**I**), and MPO (**J**). Error bars represent the SD of three independent experiments. S: colorimetric signal. PA: *Pseudomonas aeruginosa*; KP: *Klebsiella pneumoniae*; SA: *Staphylococcus aureus*; EC: *Escherichia coli*; MPO: myeloperoxidase. CFU: colony forming units. SD: standard deviation.

Figure 2 shows experiments aimed at optimizing the nanoprobes in order to obtain the highest signal-to-noise ratio in the sensor array. In these experiments, nanoprobes were modified with different concentrations of antibody and then tested against each corresponding target. A schematic illustration of the process involved in the immunodetection of pathogens and MPO is shown in Figure S5. In Figure 2A–D, the largest difference from zero was registered when antibodies were used at 12.5 µg·mL^−1^, whereas in Figure 2E the best antibody concentration was 25 µg·mL^−1^. As such, these conditions were selected for manufacturing biosensors. Figure 2F–J show that the nanoprobes yielded dose-dependent signals when analyzing samples containing their target pathogens. The limit of detection for single strip detection, defined as the first point above the signal of the blank + three standard deviations (S_blank_ +3SD), is 10^3^ CFU·mL^−1^ for *P. aeruginosa* and *E. coli* and 10^4^ CFU·mL^−1^ for *K. pneumoniae* and *S. aureus*. These LODs are well below the clinical threshold for detecting infections, which is set at 10^5^ CFU·mL^−1^ for BAS and sputum samples [21]. The LOD for MPO is 0.1 µg·mL^−1^. Experiments for detecting other bacteria, including frequent colonizers of the respiratory tract (*S. maltophilia*, *S. pneumoniae*, and *S. epidermidis*), yielded signals lower than the LOD when the pathogen was present at 10^7^ CFU mL^−1^ or less. In Figure 2F, nanoprobes against PA produced signals close to the defined LOD when exposed to other bacterial species, which could potentially lead to false-positive results. At very high concentrations, nanoprobes against *S. aureus* also yield signals above the LOD when confronted with *S. maltophilia*, *S. pneumoniae*, and *S. epidermidis* (Figure 2H). However, in both cases, signals are well below the clinical threshold for infections (10^5^ CFU mL^−1^), which eliminates the risk of a false positive. Potential non-specific interactions between nanoprobes against MPO and other antigens in the real matrix will be studied below.

**Figure 3:**
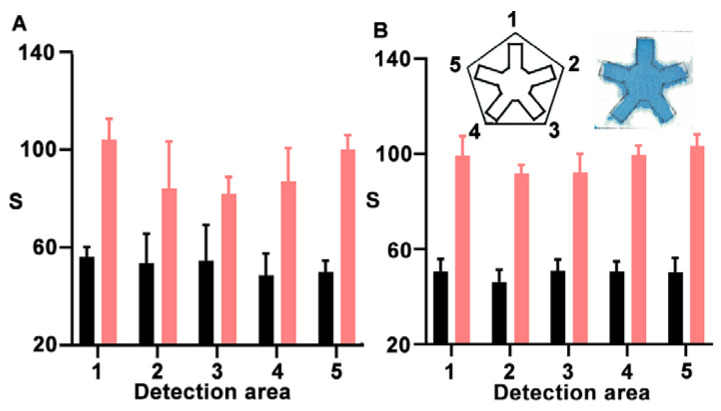
Evaluation of signal variability for the detection of BSA-biotin at 200 µg·mL^−1^ (pink) and 0 µg·mL^−1^ (black) using a pentasensor without (**A**) or with (**B**) a microfluidic system. Error bars represent the standard deviation (SD) of three independent experiments. S: colorimetric signal.

After optimizing the nanoprobes, we investigated the impact of using a microfluidic system compared to simply applying a drop of sample onto unmodified paper. To test this, we fabricated NanoArrayPAD-5 prototypes for detecting biotin-BSA as a model analyte. In Figure 3A, the pentasensor without microfluidics yields different results depending on the detection area, with an intra-sensor variability of 13.8, whereas in the wax-printed sensor (Figure 3B), this value was reduced to 7. Similarly, the inter-sensor variability was reduced from 11 to 5.8 by implementing the microfluidic system (Table S1). This demonstrates that implementing microfluidics not only facilitates the addition of solutions but also improves result reproducibility by minimizing variations related to the positioning of the reservoirs in the nanoprobe array. In the future, the variability could be reduced even further by substituting the manual printing method for an alternative that offers improved channel definition [22]. Moreover, the design has been validated for developing NanoArrayPAD-X with X between 2 and 5, and could be adapted for developing sensor arrays with higher multiplexing capabilities (Figure S6 in the Supplementary Materials).

Based on these results, we continued the design and characterization of a device capable of detecting *Pseudomonas aeruginosa* (PA), *Klebsiella pneumoniae* (KP), *Staphylococcus aureus* (SA), *Escherichia coli* (EC), and myeloperoxidase, a key biomarker of neutrophilic inflammation and highly relevant in bacterial infections [23]. The four bacterial species chosen are among the most common Gram-negative and Gram-positive pathogens responsible for lower respiratory tract infections in hospitals [24,25,26], making their identification useful to diagnose these infections. Furthermore, hospital strains of these bacteria exhibit multiple antibiotic resistance mechanisms, making them less susceptible to conventional antimicrobials [24].

Consequently, rapid and accurate identification of these pathogens is critical for choosing adequate antibiotics [1,27,28], thus providing complementary information for a correct treatment administration.

**Figure 4:**
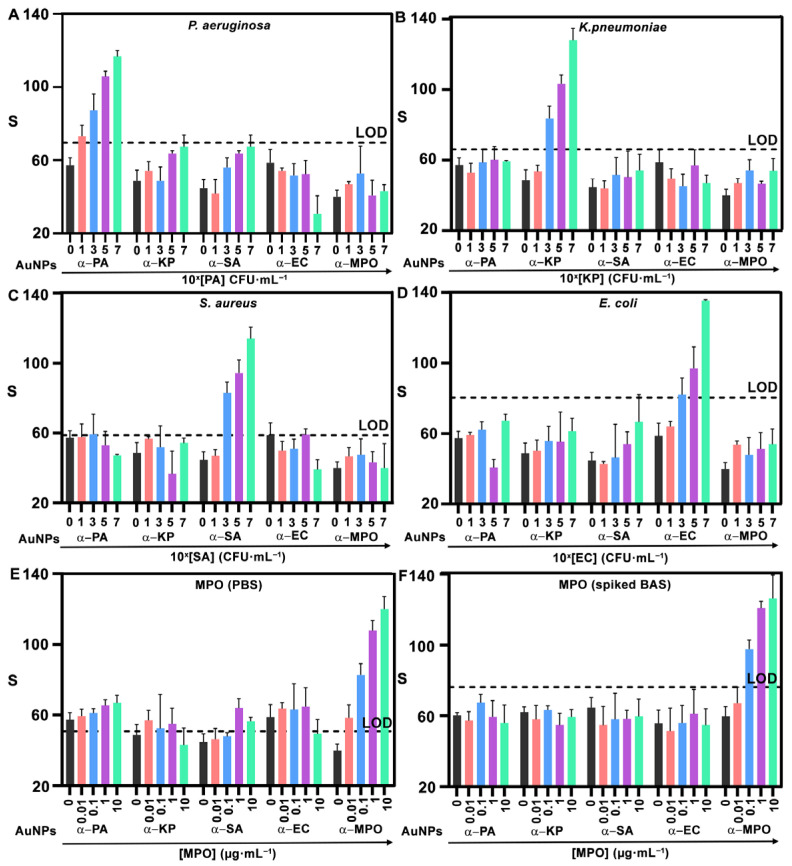
Multiplexed detection with NanoArrayPAD−5. Signals from α−PA, α−KP, α−SA, α−EC, and α−MPO nanoprobes when analyzing samples containing PA (**A**), KP (**B**), SA (**C**), and EC (**D**) suspended in PBS and MPO in PBS (**E**), or spiked into a BAS sample (**F**). In (**A**–**D**), 0, 1, 3, 5, and 7 refer to 10^0^, 10^1^, 10^3^, 10^5^, and 10^7^ CFU·mL^−1^, respectively. Error bars represent the SD of three independent experiments. Dotted lines indicate the LOD, defined as blank + 3SD. S: colorimetric signal. PA: *Pseudomonas aeruginosa*; KP: *Klebsiella pneumoniae*; SA: *Staphylococcus aureus*; EC: *Escherichia coli*; MPO: myeloperoxidase. CFU: colony forming units. BAS: bronchial aspirate sample. AuNPs: gold nanoprobes. SD: standard deviation. LOD: limit of detection.

Figure 4 shows the results obtained after testing the sensor array with increasing concentrations of all targets. Each experiment was performed in triplicate, and the dotted lines in each plot represent the limit of detection (LOD), calculated as the mean signal of the blank plus three standard deviations. All the detection areas in the sensor array yield dose-dependent signals in agreement with Figure 2. The LOD for *P. aeruginosa*, *K. pneumoniae*, and *S. aureus* is 10^3^ CFU·mL^−1^, whereas for *E. coli* it is 10^5^ CFU·mL^−1^ and for MPO it is 0.01 μg·mL^−1^. While the LOD for *P. aeruginosa*, *K. pneumoniae*, and *S. aureus* decreased or remained stable, it increased for *E. coli* (from 10^3^ CFU·mL^−1^, Figure 2I, to 10^5^ CFU·mL^−1^, Figure 4D). Previous studies have shown that bacterial retention on paper substrates varies depending on the microorganism. This may change in the concentration of bacteria as they travel through the paper-based microfluidic channels, thus affecting the concentration of bacteria trapped in the detection zone and influencing assay performance [29,30,31]. Such effects could account for the observed increase in the LOD for *E. coli*. Nanoprobes against bacteria do not recognize other cells; that is, all signals for detecting bacteria other than the target are below the LOD. However, the same nanoprobes yield signals above the LOD when detecting MPO in Figure 4E. This is ascribed to the cationic nature of this protein, which facilitates non-specific interactions with negatively charged nanoprobes [32,33]. We hypothesized that these interferences would diminish in the real matrix, which contains abundant proteins that may block non-specific interactions. To demonstrate this, an additional experiment with MPO spiked into liquefied BAS samples was performed (Figure 4F). This yielded a higher LOD (0.01 μg·mL^−1^) but no false positives in any of the other detection areas. These experiments stress the relevance of testing the performance of biomarker detection in real matrices, especially when dealing with positively charged proteins. The performance of the sensing platform was further evaluated in the presence of samples containing a mixture of multiple target pathogens. Results in Figure S7 confirmed that different bacterial species can be simultaneously detected with reliable performance. 

**Figure 5:**
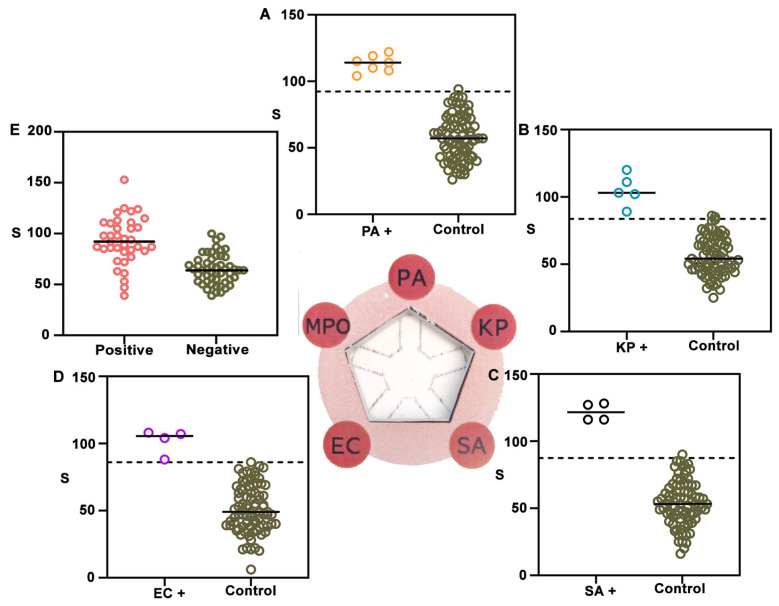
NanoArrayPAD−5 detection of bacteria and biomarkers in liquefied BAS samples. Colorimetric signals from nanoprobes conjugated with antibodies against PA (**A**), KP (**B**), SA (**C**), EC (**D**), and MPO (**E**) were measured in 87 samples. Signal distributions for isolated target bacteria are shown in orange (PA), blue (KP), black (SA), and purple (EC), while controls (non-target pathogens, mixed flora, or other pathogens) are shown in green. Central bars represent means and dotted lines indicate the detection cut-off (mean + 2 SD). α-MPO signals (**E**) were higher in culture-positive samples (red). S: colorimetric signal. PA: *Pseudomonas aeruginosa*; KP: *Klebsiella pneumoniae*; SA: *Staphylococcus aureus*; EC: *Escherichia coli*; MPO: myeloperoxidase. CFU: colony forming units. BAS: bronchial aspirate sample. SD: standard deviation.

Finally, NanoArrayPAD-5 was validated with real patient samples. To this end, we tested a collection of bronchial aspirate samples (BAS) obtained from intubated patients in the ICU of Son Llàtzer University Hospital (Figure 5). Out of 87 samples, PA was isolated in 7, KP in 5, SA in 4, and EC in 4. Additionally, 12 samples contained mixed flora and 39 showed no bacterial growth. The remaining samples contained other bacterial species or fungi. A detailed breakdown of the pathogens present in each sample is provided in Table S2 from Section S10 of the Supplementary Materials. For bacterial detection, data is divided in two categories depending on whether the sample contained the target pathogen at a concentration ≥10^5^ CFU·mL^−1^ (bacteria+) or not (control). Dotted lines indicate cut-off values, calculated as the mean signal from control samples plus two times the SD. The target bacterial species was always successfully identified in Figure 5A–D, yielding a true positive rate of 100%. False positives were rare (1.25% for PA, 2.44% for KP, 1.2% for *S. aureus*, and 1.2% for *E. coli*). These findings underscore the high specificity and analytical reliability of the platform for multiplexed bacterial detection despite potential signal inhibition in complex samples. With regard to the MPO detection area, the samples were categorized as culture-positive (black) or culture-negative (green). In Figure 5E, the mean signal intensity was significantly higher in culture-positive samples compared to -negative ones. Using a cut-off value of 71.5, culture-positive samples were identified with a sensitivity of 72.5% and a specificity of 84.2%. Similar values have been reported when using MPO to identify pulmonary infections (Table S3). However, it should be noted that the sensitivity and specificity may change depending on the cut-off value, and that this value could be fine-tuned to either maximize sensitivity or specificity (Table S4). Here, we propose that MPO could be used to screen patients that could develop VAP due to the presence of potentially pathogenic microorganisms in their lower respiratory tract.

In future work, the implementation of sandwich immunoassays could enhance both the selectivity and sensitivity of the assay. The use of monoclonal capture antibodies would improve target analyte retention on the paper matrix while minimizing non-specific interactions, as monoclonal antibodies recognize a single epitope of the analyte [34]. Furthermore, combining sandwich immunoassays with electrochemical detection has been reported as an effective strategy to further increase the sensitivity and specificity of immunoassays [35,36]. If the colorimetric signal generation mechanism is maintained, a rapid quantification via mobile app could facilitate the implementation of NanoArrayPAD at the POC.

## 4. Conclusions

In conclusion, we have introduced NanoArrayPAD−5, an innovative paper-based multisensor platform that combines microfluidic paper technology with arrays of nanoprobes for the simultaneous detection of four pathogens and one biomarker in respiratory samples. Initial validation in buffered solutions and spiked samples confirm the multiplexing capability of the platform and specificity of the nanoprobes, achieving limits of detection at or below clinical thresholds for infection diagnosis. While MPO exhibits non-specific interactions due to its cationic nature, these are effectively mitigated when spiked into the real matrix. Diagnostic performance was validated using 87 BASs from intubated patients. Despite the inherent complexity and variability of sputum matrices, NanoArrayPAD−5 demonstrates low false-positive rates for all pathogens, and sensitivity and specificity values for MPO within clinically accepted ranges. The total turnaround time (including sample processing) is 25 min. Moreover, the number of targets that are simultaneously detected can be easily fine-tuned by redesigning the microfluidic system and the geometry of the array. These features make NanoArraPAD-X a promising candidate for helping physicians diagnose respiratory diseases rapidly and at the point of care.

## Data Availability

Data will be made available on request.

## Supplementary Materials

The following supporting information can be downloaded at: https://www.mdpi.com/article/10.3390/bios15110715/s1, Figure S1: Parts of the housing cassette of NanoArrayPAD−X. From left to right: cassette parts and reservoir plugs corresponding to NanoArrayPAD 2, 3, 4, and 5. Items framed in blue represent 3D-printed components, while those framed in black are commercially sourced parts. Figure S2: Wax printing in paper strips for NanoArrayPAD−5 (A) and assembly of the whole NanoArrayPAD−5 cassette (B). Figure S3: Evaluation of color intensity variation (S) across different sample volumes (10, 20, 30, and 40 µL) for four NanoArrayPAD devices. (A) NanoArrayPAD−2. (B) NanoArrayPAD−3. (C) NanoArrayPAD−4. (D) NanoArrayPAD−5. Each bar represents the mean in the color intensity quantified in each detection channel. Error bars represent the standard deviation of three independent experiments. Figure S4: UV–Vis spectra of gold nanoprobes (A) and image analysis using ImageJ (B). In (A), spectra ranging from 400 to 800 nm are shown for citrate-stabilized nanoprobes (black), PEGylated nanoprobes (green), avidin-decorated nanoprobes (orange), and antibody-coated gold nanoprobes (pink) (A). The localized surface plasmon resonance (LSPR) is also indicated. In (B) the selected region of interest (ROI) used for quantification in ImageJ is highlighted in red. Both the raw values and the colorimetric signal S are indicated. A: absorbance; λ: wavelength; S: signal. Figure S5: Schematic representation of the process for detection of bacteria (A) and MPO (B) using gold nanoprobes. First, bacteria and MPO are retained within the paper matrix. The antibody-decorated gold nanoprobes are then transferred from the reservoir, allowing specific interactions to occur between the probes and their target analytes. Finally, unbound nanoprobes are washed away, and the resulting colorimetric signal is revealed. Table S1: Intra- and inter-sensor variability in the proposed array configurations. Figure S6: Evaluation of signal variability across different NanoArrayPAD−X designs for the detection of BSA-biotin at 200 µg·mL^−1^ (pink) and 0 µg·mL^−1^ (black). Images of the NanoArrayPAD−X devices are displayed above each panel, with the hydrophilic channels filled with blue ink. Devices with X = 2 (A), 3 (B), 4 (C). Error bars represent the standard deviation (SD) from three replicates (see also Table S1). Figure S7: Impact of the simultaneous presence of two target bacteria on the detection performance of NanoArrayPAD-5. Signals obtained with anti-PA (orange), anti-KP (blue), anti-SA (black), anti-EC (pink), and anti-MPO (red) are shown. The bacterial suspensions and the concentration of each pathogen are indicated in each graph. Error bars represent the SD of three independent experiments. S: colorimetric signal. PA: *Pseudomonas aeruginosa*; KP: *Klebsiella pneumoniae*; SA: *Staphylococcus aureus*; EC: *Escherichia coli*; MPO: myeloperoxidase. CFU: colony forming units. SD: standard deviation. Table S2: Microbiology reported results in culture-positive BAS samples. Table S3: Reported sensitivity and specificity of MPO as a biomarker of inflammation across various diseases. Table S4: Sensitivity and specificity of MPO as a biomarker for infection diagnosis depending on the cut-off values, references [37,38,39,40].

## Abbreviations

The following abbreviations are used in this manuscript:

VAP	Ventilator-associated pneumonia
ICU	Intensive Care Unit
3D-µPAD	Three-dimensional microfluidic paper-based analytical device
µPAD	Microfluidic paper-based analytical device
PA	*Pseudomonas aeruginosa*
KP	*Klebsiella pneumoniae*
SA	*Staphylococcus aureus*
EC	*Escherichia coli*
MPO	Myeloperoxidase
BSA	Bovine serum albumin
PBS	Phosphate-buffered saline
PBST	Phosphate-buffered saline with 0.1% TWEEN 20
AuNPs	Gold nanoprobes
PI	Pixel intensity
BAS	Bronchial aspirate sample
SD	Standard deviation
