# Supplementary material for: NanoArrayPAD−X: Nanoprobe Array and 3D-µPAD for the Simultaneous Detection of Respiratory Pathogens and Biomarkers at the Point of Care
Source: Biosensors (Basel). 2025 Oct 28;15(11):715. doi: 10.3390/bios15110715 (PMC12650703; doi:10.3390/bios15110715)
Supplement: Supplementary file 1 [file biosensors-15-00715-s001.zip › biosensors-3903885-supplementary.docx]

**Supporting information**

**NanoArrayPAD−X: Nanoprobe Array and 3D-µPAD for the Simultaneous Detection of Respiratory Pathogens and
Biomarkers at the Point of Care**

Andreu Vaquer ^1,2,^*, Francisco M. Bouzada ^1^, Sofia Tejada ^1^, Antonio Clemente ^3,4^, Antonia Socias ^1,5^,
Maria Aranda ^1,5^, Alberto del Castillo ^1,5^, Joana Mena ^1,5^, Maria Montaner ^6^, Rocío Rodríguez ^6^,
Estrella Rojo-Molinero ^4,6^, Antonio Oliver ^4,6^, Marcio Borges ^1,5^ and Roberto de la Rica ^1,4,^*

^1^ Multidisciplinary Sepsis Group, Health Research Institute of Balearic Islands (IdISBa), Son Espases University Hospital, 07120 Palma de Mallorca, Spain

^2^ Department of Chemistry, University of the Balearic Islands, 07122 Palma de Mallorca, Spain

^3^ Group of Innovation in Immunopathology of Infections (GTERi), Health Research Institute of the Balearic Islands (IdISBa), 07120 Palma de Mallorca, Spain

^4^ CIBER de Enfermedades Infecciosas (CIBERINFEC), Instituto de Salud Carlos III, 28029 Madrid, Spain

^5^ Multidisciplinary Sepsis Unit, Son Llàtzer University Hospital, 07198 Palma de Mallorca, Spain

^6^ Microbiology Department, Health Research Institute of Balearic Islands (IdISBa), Son Espases University Hospital, 07120 Palma de Mallorca, Spain

* Correspondence: andreu.vaquer@idisba.es (A.V.); roberto.delarica@idisba.es (R.d.l.R.)

**Table of contents**

[Section S1. Cassette parts of NanoArrayPAD−X 3](#_Toc211421061)

[3](#_Toc211421062)

[Section S2. Wax printing and assembly of NanoArrayPAD−X 4](#_Toc211421063)

[Section S3. Synthesis of AuNPs and modification with avidin and antibodies 5](#_Toc211421064)

[Section S4. Sample volume optimization for NanoArrayPAD−X 6](#_Toc211421065)

[Section S5. UV-Vis spectrometry of nanoprobes and densitometric analysis of colorimetric signals using ImageJ 7](#_Toc211421066)

[Section S6. Schematic representation of the interaction of nanoprobes with target analytes 8](#_Toc211421067)

[Section S7. Intra- and inter-sensor variability in the proposed array configurations 9](#_Toc211421068)

[Section S8. Performance of different configurations of NanoArrayPAD−X 10](#_Toc211421069)

[Section S9. Impact of Coexisting Target Bacteria on the Analytical Performance of NanoArrayPAD-5 11](#_Toc211421070)

[Section S10. Isolated pathogens in the BAS samples applied to NanoArrayPAD−5 12](#_Toc211421071)

[Section S11. Sensitivity and specificity of MPO as a biomarker for disease prediction 13](#_Toc211421072)

[Section S12. Impact of cut-off values on MPO sensitivity and specificity as a biomarker for infection diagnosis. 14](#_Toc211421073)

[References 15](#_Toc211421074)

Section S1. Cassette parts of NanoArrayPAD−X


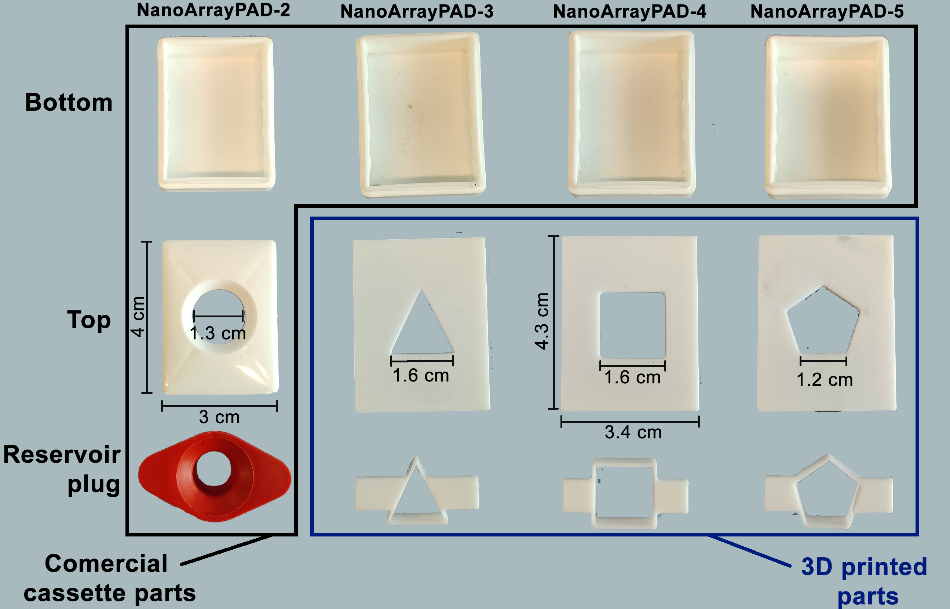


**Figure S1.** Parts of the housing cassette of NanoArrayPAD−X. From left to right: cassette parts and reservoir plugs corresponding to NanoArrayPAD 2, 3, 4, and 5. Items framed in blue represent 3D-printed components, while those framed in black are commercially sourced parts.


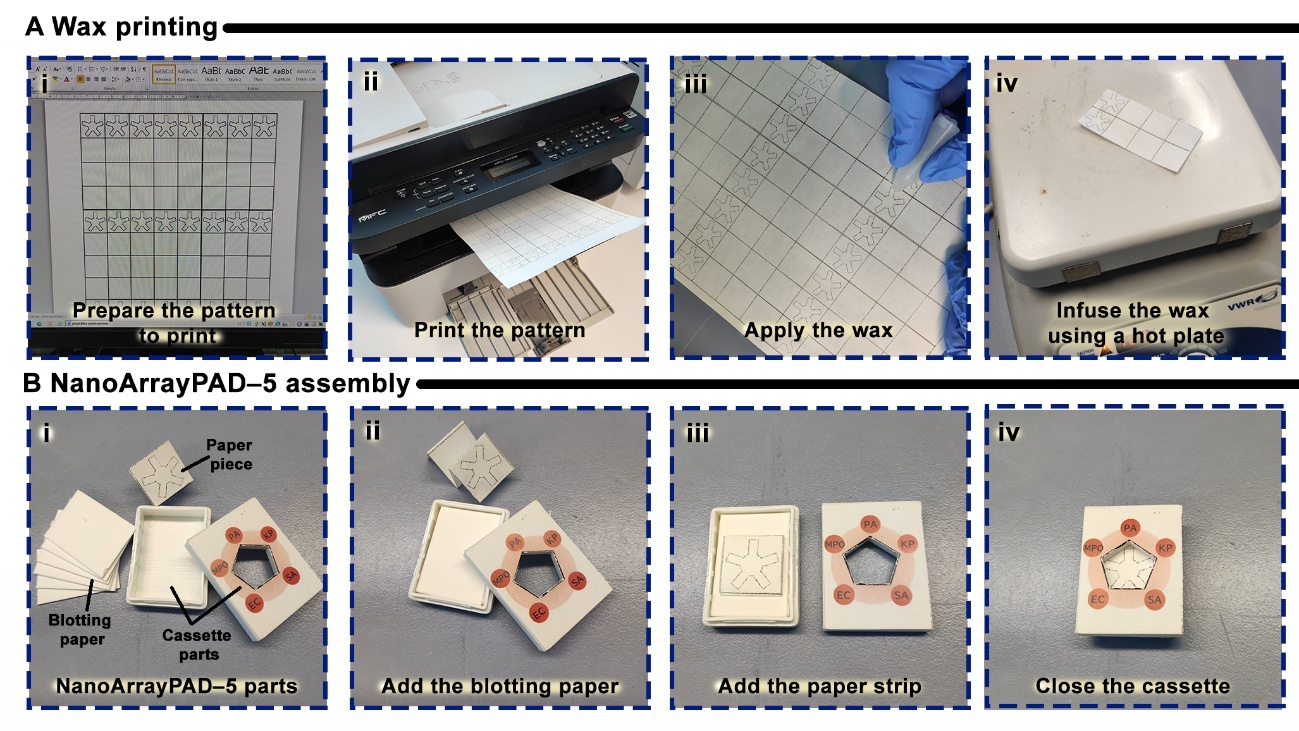
Section S2. Wax printing and assembly of NanoArrayPAD−X

**Figure S2.** Wax printing in paper strips for NanoArrayPAD−5 (A) and assembly of the whole NanoArrayPAD−5 cassette (B).

Section S3. Synthesis of AuNPs and modification with avidin and antibodies

Gold nanoprobes (AuNPs) approximately 40 nm in diameter were synthesized in a 250 mL solution using a citrate reduction method. To prepare targeted nanoprobes for targeted bacteria and MPO, the AuNP surfaces were covalently functionalized with avidin and subsequently conjugated with biotinylated specific antibodies. In brief, 49 mg of gold chloride and 57 mg of sodium citrate were dissolved in 250 mL of boiling water, stirred, and allowed to react for 15 minutes. The resulting nanoprobes were then pegylated by adding 0.1 mM SH-PEG-COOH and stirring overnight.

Afterward, the nanoprobes were concentrated 250-fold by centrifugation (8 minutes at 9000 rpm) and washed five times with Mili-Q water. For covalent functionalization with avidin, the pegylated AuNPs were resuspended in 0.5 M MES buffer (pH 5.5) containing 10 mM EDC and sulfo-NHS. Following 30-minute incubation, the particles were centrifuged and resuspended in a PBS solution containing 0.5 mg·mL^-1^ of avidin, and then incubated overnight at 4°C. The next day, a blocking solution of 0.1 M glycine and 10 mg·mL^-1^ BSA in phosphate buffer (pH 7) was added for 30 minutes to cap unreacted sites. The particles were washed three times with PBST buffer.

To generate antibody-functionalized AuNPs (Ab-AuNPs), 100 µL of the avidin-coated AuNPs were incubated with 10 µL of biotinylated polyclonal antibodies for one hour. Next, 10 µL of 0.1 mM PEG-biotin was added for 30 minutes to block any remaining biotin-binding sites. Unbound components were removed by centrifugation (three times at 7000 rpm for 6 minutes), and the particles were resuspended in PBST. Finally, the Ab-AuNPs were stored in 100 µL of PBST at 4°C until further use.

Section S4. Sample volume optimization for NanoArrayPAD−X

A drop of water-based blue ink was applied to the inlet of each NanoArrayPAD device. The ink was allowed to spread to the detection channels, after which the paper strip was removed from the cassette and scanned. Pixel intensity in each detection channel was then quantified using ImageJ. All experiments were performed in triplicate, testing sample volumes of 10, 20, 30, and 40 µL. The optimal sample volume was determined as the one that showed the most reproducible color intensity (S), both within each replicate and between different replicates. Based on this criterion, a volume of 10 µL was selected for NanoArrayPAD−2. For NanoArrayPAD−3 and NanoArrayPAD−4, 30 µL was selected as the volume with lower variability. Finally, for NanoArrayPAD−5, 20 µL was selected as the most suitable volume.


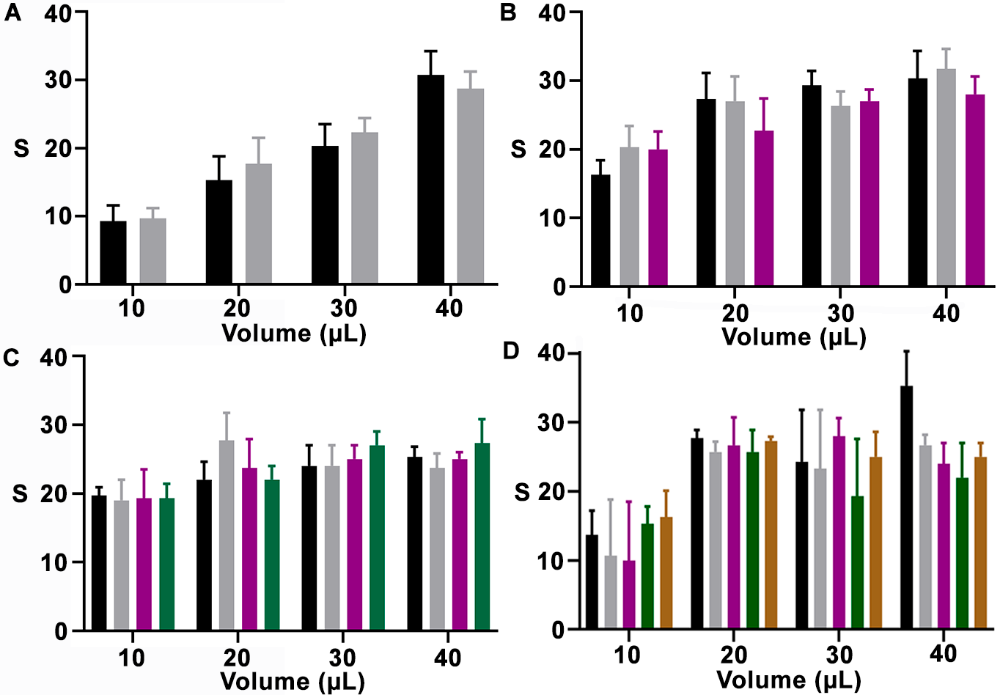


**Figure S3.** Evaluation of color intensity variation (S) across different sample volumes (10, 20, 30, and 40 µL) for four NanoArrayPAD devices. (A) NanoArrayPAD−2. (B) NanoArrayPAD−3. (C) NanoArrayPAD−4. (D) NanoArrayPAD−5. Each bar represents the mean in the color intensity quantified in each detection channel. Error bars represent the standard deviation of three independent experiments.

Section S5. UV-Vis spectrometry of nanoprobes and densitometric analysis of colorimetric signals using ImageJ


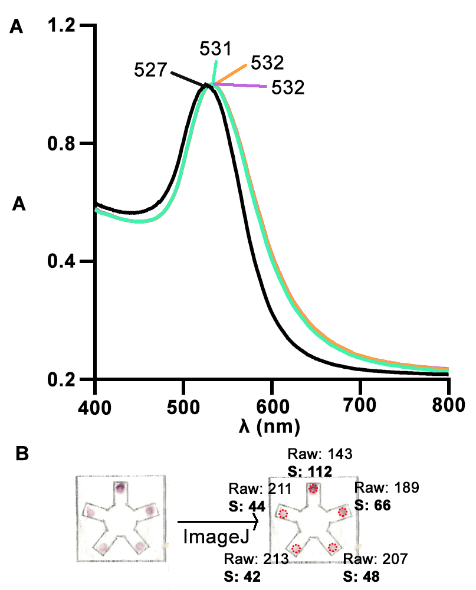


**Figure S4. UV–Vis spectra of gold nanoprobes (A) and image analysis using ImageJ (B)**. In (A), spectra ranging from 400 to 800 nm are shown for citrate-stabilized nanoprobes (black), PEGylated nanoprobes (green), avidin-decorated nanoprobes (orange), and antibody-coated gold nanoprobes (pink) (A). The localized surface plasmon resonance (LSPR) is also indicated. In (B) the selected region of interest (ROI) used for quantification in ImageJ is highlighted in red. Both the raw values and the colorimetric signal S are indicated. A: absorbance; λ: wavelength; S: signal.

Section S6. Schematic representation of the interaction of nanoprobes with target analytes


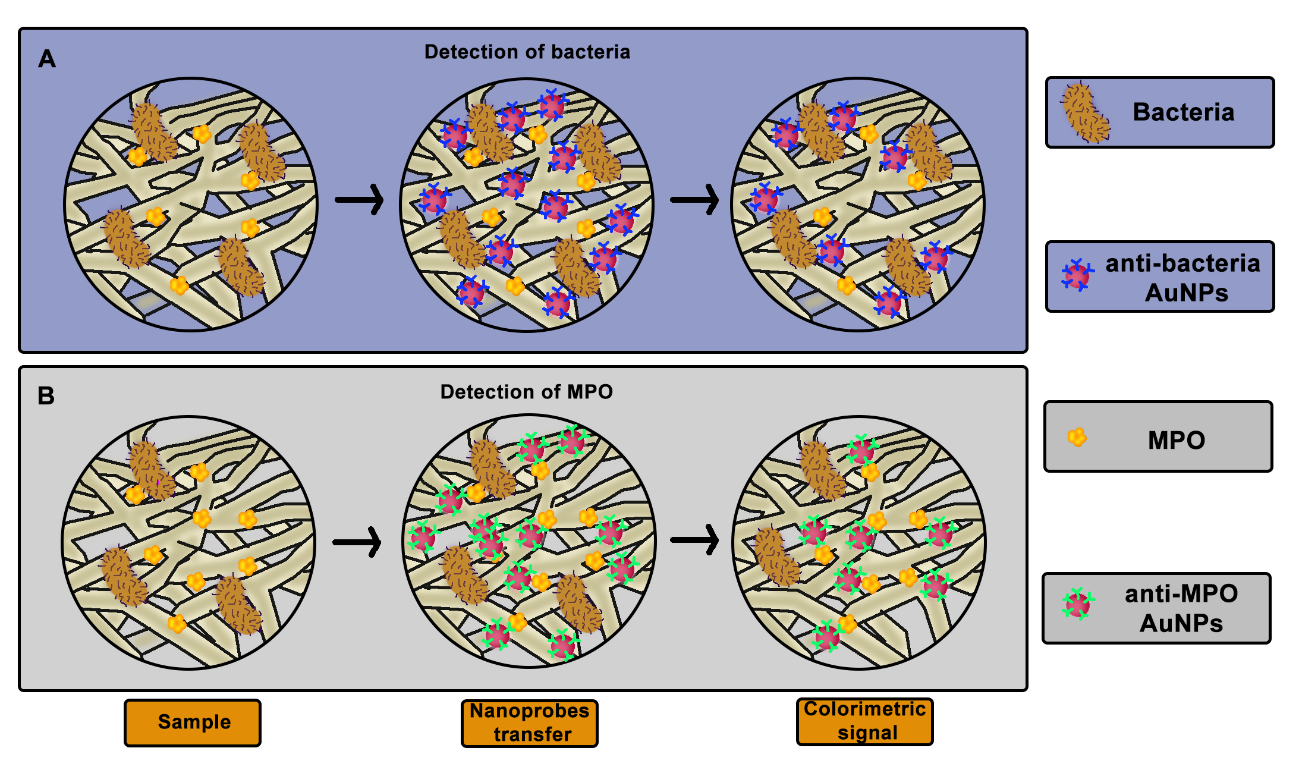


**Figure S5. Schematic representation of the process for detection of bacteria (A) and MPO (B) using gold nanoprobes**. First, bacteria and MPO are retained within the paper matrix. The antibody-decorated gold nanoprobes are then transferred from the reservoir, allowing specific interactions to occur between the probes and their target analytes. Finally, unbound nanoprobes are washed away, and the resulting colorimetric signal is revealed.

Section S7. Intra- and inter-sensor variability in the proposed array configurations

**Table S1.**

Table S1. Intra- and inter-sensor variability in the proposed array configurations

| **Nº spots** | **SD**  **intra-sensor**  **C-** | **SD**  **Intra-sensor**  **C+** | **SD**  **inter-sensor**  **C-** | **SD**  **Inter-sensor**  **C+** |
| --- | --- | --- | --- | --- |
| 2 | 3.8 | 6.7 | 3.1 | 6.1 |
| 3 | 4.9 | 8.4 | 5.9 | 8.3 |
| 4 | 8.9 | 3.9 | 9.4 | 3.5 |
| 5 | 4.6 | 7.0 | 5.0 | 5.8 |
| 5 (no wax) | 8.8 | 13.8 | 8.9 | 11 |

C+ refers to a positive control ([BSA-biotin]: 200 µg·mL⁻¹). C- refers to a negative control ([BSA-biotin] of 0 µg·mL⁻¹)

Section S8. Performance of different configurations of NanoArrayPAD−X


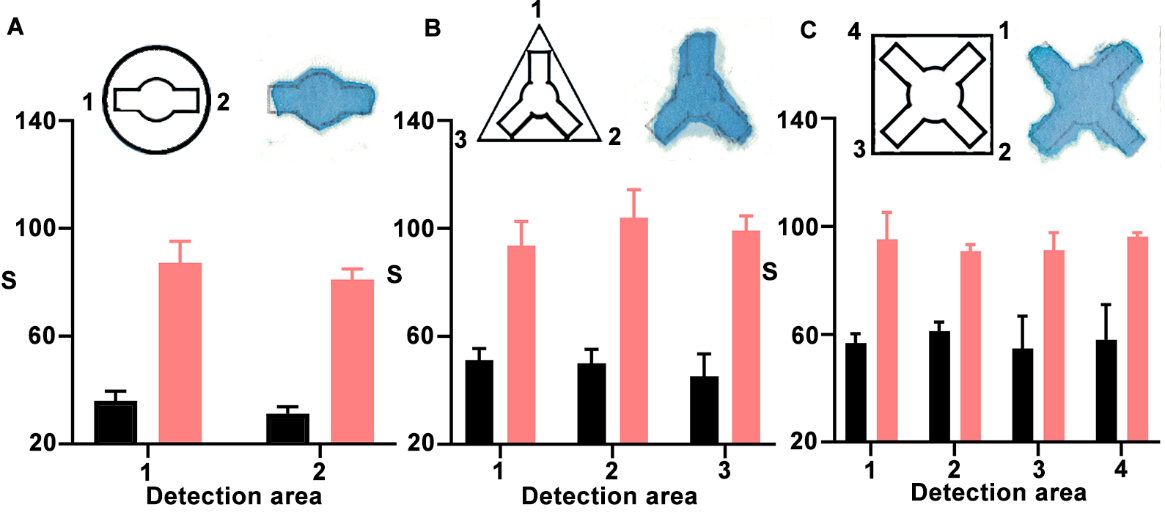


**Figure S6.** Evaluation of signal variability across different NanoArrayPAD−X designs for the detection of BSA-biotin at 200 µg·mL⁻¹ (pink) and 0 µg·mL⁻¹ (black). Images of the NanoArrayPAD−X devices are displayed above each panel, with the hydrophilic channels filled with blue ink. Devices with X = 2 (A), 3 (B), 4 (C). Error bars represent the standard deviation (SD) from three replicates (see also Table S1).

Section S9. Impact of coexisting target bacteria on the analytical performance of nanoarraypad-5


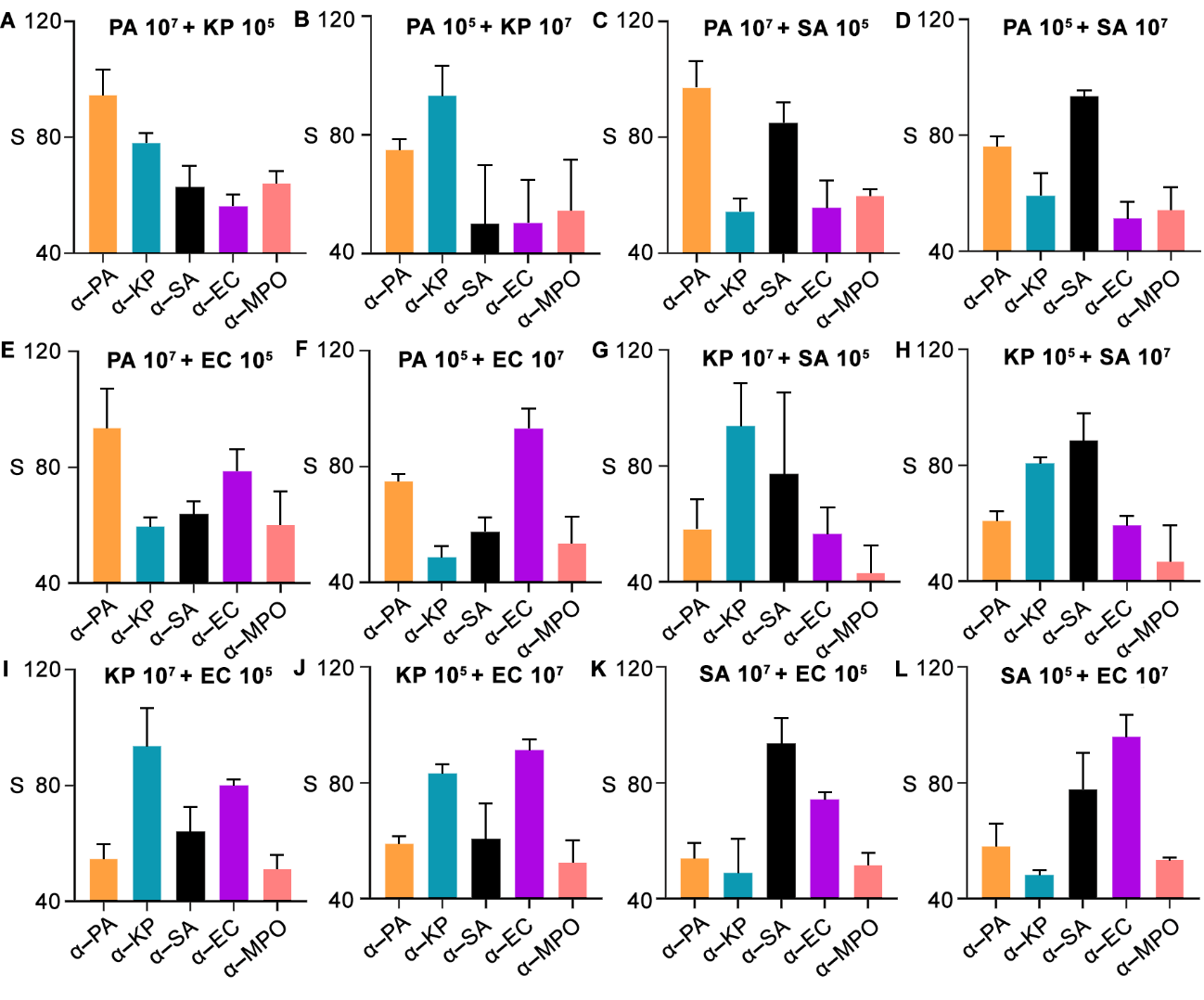


**Figure S7. Impact of the simultaneous presence of two target bacteria on the detection performance of NanoArrayPAD-5.** Signals obtained with anti-PA (orange), anti-KP (blue), anti-SA (black), anti-EC (pink), and anti-MPO (red) are shown. The bacterial suspensions and the concentration of each pathogen are indicated in each graph. Error bars represent the SD of three independent experiments. S: colorimetric signal. PA: *Pseudomonas aeruginosa*; KP: *Klebsiella pneumoniae*; SA: *Staphylococcus aureus*; EC: *Escherichia coli*; MPO: myeloperoxidase. CFU: colony forming units. SD: standard deviation.

Section S10. Isolated pathogens in the BAS samples applied to NanoArrayPAD−5

**Table S2**. Microbiology reported results in culture-positive BAS samples

| **Sample number** | **Infection** |
| --- | --- |
| **1** | *S. aureus* |
| **2** | *S. epidermidis* |
| **3** | *K. pneumoniae + P. mirabilis* |
| **4** | *K. pneumoniae* |
| **5** | *K. pneumoniae + P. mirabilis* |
| **6** | *K. pneumoniae + P. mirabilis* |
| **7** | *S. aureus* |
| **8** | *S. maltophilia* |
| **9** | *R. mucilaginosa* |
| **10** | *K. oxytoca + E. coli* |
| **11** | *S. epidermidis* |
| **12** | *S. epidermidis* |
| **13** | *S. epidermidis* |
| **14** | *C. albicans* |
| **15** | *S. aureus* |
| **16** | *S. parasanginis* |
| **17** | *S. aureus* |
| **18** | *S. liquefaciens* |
| **19** | *S. epidermidis + C. tropicalis* |
| **20** | *S. maltophilia* |
| **21** | *Candida sp.* |
| **Sample number** | **Infection** |
| **22** | *C. albicans* |
| **23** | *C. albicans* |
| **24** | *S. epidermidis* |
| **25** | *Mixed flora* + *S. epidermidis* |
| **26** | *E. coli* |
| **27** | *E. coli* |
| **28** | *P. aeruginosa* |
| **29** | *P. aeruginosa* |
| **30** | *S. maltophilia + E. coli* |
| **31** | *P. aeruginosa* |
| **32** | *P. aeruginosa* |
| **33** | *K. pneumoniae* |
| **34** | *P. aeruginosa* |
| **35** | *P. aeruginosa* |
| **36** | *P. aeruginosa* |
|  |  |
|  |  |
|  |  |
|  |  |
|  |  |

Section S11. Sensitivity and specificity of MPO as a biomarker for disease prediction

**Table S3.** Reported sensitivity and specificity of MPO as a biomarker of inflammation across various diseases.

| **Disease** | **Sample** | **Cut-off** | **Sensitivity (%)** | **Specificity (%)** | **Ref** |
| --- | --- | --- | --- | --- | --- |
| **Pneumonia** | **BAS** | **71.5 (S)** | **72.5** | **84.2** | **Current research** |
| **Cystic fibrosis** | BAL | 0.52 ng·mL^-1^ | 64 | 71 | [1] |
| **COPD** | Induced sputum | 6.24 ng·mL^-1^ | 58.3 | 100 | [2] |
| **T-COPD** | Serum | 63.4 mmol·mL^-1^ | 70.5 | 80.9 | [3] |
| **Asthma** | Serum | 2600 pg·mL^-1^ | 84.6 | 100 | [4] |
| **Severe asthma** | Serum | 3400 pg·mL^-1^ | 85.9 | 86.4 | [4] |

*BAS: Bronchial aspirate sample; BAL: bronchoalveolar lavage; COPD: Chronic Obstructive Pulmonary Disease; Treated Chronic Obstructive Pulmonary Disease.

Section S12. Impact of cut-off values on MPO sensitivity and specificity as a biomarker for infection diagnosis.

**Table S4.** Sensitivity and specificity of MPO as a biomarker for infection diagnosis depending on the cut-off values.

| **Cut-off value** | **Sensitivity (%)** | **Specificity (%)** |
| --- | --- | --- |
| 40.5 | 1.9 | 97.4 |
| 51 | 19.6 | 92.1 |
| 60.5 | 37.3 | 89.5 |
| **71.5** | **72.5** | **84.2** |
| 80 | 82.3 | 76.3 |
| 90.5 | 84.1 | 50 |
| 102.5 | 100 | 34.2 |

References

1. Horati, H.; Margaroli, C.; Chandler, J.D.; Kilgore, M.B.; Manai, B.; Andrinopoulou, E.-R.; Peng, L.; Guglani, L.; Tiddens, H.A.M.W.; Caudri, D.; et al. Key Inflammatory Markers in Bronchoalveolar Lavage Predict Bronchiectasis Progression in Young Children with CF. *Journal of Cystic Fibrosis* **2024**, *23*, 450–456, doi:10.1016/j.jcf.2024.01.002.

2. Zhu, T.; Li, S.; Wang, J.; Liu, C.; Gao, L.; Zeng, Y.; Mao, R.; Cui, B.; Ji, H.; Chen, Z. Induced Sputum Metabolomic Profiles and Oxidative Stress Are Associated with Chronic Obstructive Pulmonary Disease (COPD) Severity: Potential Use for Predictive, Preventive, and Personalized Medicine. *EPMA Journal* **2020**, *11*, 645–659, doi:10.1007/s13167-020-00227-w.

3. Jiang, E.; Fu, Y.; Wang, Y.; Ying, L.; Li, W. The Role and Clinical Significance of Myeloperoxidase (MPO) and TNF-α in Prognostic Evaluation of T-COPD. *BMC Pulmonary Medicine* **2025**, *25*, 192, doi:10.1186/s12890-025-03655-4.

4. Hassan, K.A.-E.; Kinawy, S.A.-E.; Mahmoud, E.M. The Role of Serum Myeloperoxidase in Prediction of Severe Bronchial Asthma. *International Journal of Medical Arts* **2023**, *5*, 3373–3378, doi:10.21608/ijma.2023.220098.1723.
